# 下调βⅢ-tubulin逆转肺腺癌A549/Taxol细胞株紫杉醇耐药

**DOI:** 10.3779/j.issn.1009-3419.2014.08.01

**Published:** 2014-08-20

**Authors:** 银玲 禚, 其森 郭

**Affiliations:** 250002 济南，山东省职业病医院内科 Department of Internal Medicine, Shandong Province Hospital of Occupational Diseases, Jinan 250002, China

**Keywords:** 肺肿瘤, A549/Taxol, RNA干扰, βⅢ-tubulin, 紫杉醇, Lung neoplasms, A549/Taxol, RNA interference, βⅢ-tubulin, Taxol

## Abstract

**背景与目的:**

化疗耐药导致肿瘤很快复发和/或转移, 是目前肺癌死亡的主要原因之一。β-tubulin是抗微管药物的主要细胞靶点。已有的研究证明:βⅢ-tubulin高表达与非小细胞肺癌(non-small cell lung cancer, NSCLC)耐药有关。利用RNA干扰技术沉默耐紫杉醇A549细胞(A549/Taxol)中*βⅢ-tubulin*基因表达, 探讨靶基因下调后对化疗药物紫杉醇的敏感性的变化以及细胞周期和细胞凋亡情况。

**方法:**

构建靶向βⅢ-tubulin的siRNA, 以脂质体为载体介导βⅢ-tubulin siRNA转染A549/Taxol细胞, 利用qRT-PCR检测细胞内βⅢ-tubulin mRNA的变化情况, 并筛选出最佳干扰序列; Western blot法检测A549/Taxol细胞内βⅢ-tubulin蛋白表达的变化; MTT法检测转染后细胞株对紫杉醇敏感性的变化; 流式细胞仪检测细胞周期和细胞凋亡的变化。

**结果:**

实时荧光qRT-PCR法显示转染后细胞株靶基因水平较对照组降低, 其中βⅢ-tubulin siRNA-1序列抑制率最高为(87.73±4.87)%(*P* < 0.01);Western blot显示转染后靶蛋白水平较对照组明显降低; MTT法表明紫杉醇处理转染后细胞株的细胞抑制率较对照组明显增加(51.77±4.60)%(*P* < 0.01);细胞凋亡显示βⅢ-tubulin siRNA+Taxol组细胞早期凋亡率较对照组明显增加(*P* < 0.01), 两者的差异有统计学意义; 细胞周期检测结果显示紫杉醇处理组的G_2_/M期细胞百分率高于对照组, 且转染后紫杉醇处理组的细胞晚期凋亡率较对照组增加。

**结论:**

βⅢ-tubulin表达下调明显提高A549/Taxol细胞株对Taxol的敏感性。

## Abstract (en)

**Background and objective:**

Chemotherapy drug resistance is the primary causes of death in patients with pulmonary carcinoma which make tumor recurrence or metastasis.β-tubulin is the main cell targets of anti-microtubule drug.Increased expression of βⅢ-tubulin has been implicated in non-small cell lung cancer (NSCLC) cell lines.To explore the relationship among the expression level of βⅢ-tubulin and the sensitivity of A549/Taxolcell lines to Taxol and cell cycles and cell apoptosis by RNA interference-mediated inhibition of βⅢ-tubulin in A549/Taxol cells.

**Methods:**

Three pairs of siRNA targetd βⅢ-tubulin were designed and prepared, which were transfected into A549/Taxol cells using Lipofectamine^TM^ 2000.We detected the expression of βⅢ-tubulin mRNA using Real-time fluorescence qRT-PCR.Tedhen we selected the most efficient siRNA by the expression of βⅢ-tubulin mRNA in transfected group.βⅢ-tubulin protein level were mesured by Western blot.The taxol sensitivity in transfected group were evaluated by MTT assay.And the cell apoptosis and cell cycles were determined by flow cytometry.

**Results:**

βⅢ-tubulin mRNA levels in A549/Taxol cells were significantly decreased in transfected grop by Real-time qRT-PCR than control groups.And βⅢ-tubulin siRNA-1 sequence showed the highest transfection efficiency, which was (87.73±4.87)% (*P* < 0.01);Western blot results showed that the expressional level of BⅢ tublin protein was significantly down-reulated in the transfectant cells than thant in the control cells.By MTT assay, we showed that the inhibition ratio of Taxol to A549/Taxol cells transfeced was higher than that of control group (51.77±4.60)% (*P* < 0.01).The early apoptosis rate of A549/Taxol cells in transfected group were significantly higher than that of control group (*P* < 0.01);G_2_/M content in taxol group obviously increased than untreated samples by the cell cycle (*P* < 0.05).

**Conclusion:**

βⅢ-tubulin down-regulated significantly sensitized NSCLC A549/Taxol cells to Paclitaxel.

肺癌是当今世界上最常见的癌症, 每年约有100万患者被诊断为肺癌, 并且是癌症相关死亡的主要原因^[1]^。非小细胞肺癌(non-small cell lung cancer, NSCLC)中约半数以上的患者在诊断时已属晚期, 因此化疗是最主要的治疗方案^[2]^。在临床应用中, 铂类联合化疗已经成为NSCLC的标准化疗方案, 然而由于肿瘤耐药的出现, 使得绝大部分肿瘤很快出现复发和/或转移。从而限制了许多化疗药物例如紫杉醇、多西他赛和长春瑞滨等微管结合药物在NSCLC治疗的临床应用^[3, 4]^。

微管(microtubule)是构成细胞骨架的主要成分, 微管蛋白分为α、β两个亚型, 由α-tubulin和β-tubulin形成的异二聚体组装成的多聚体, 具有维持细胞形态, 进行物质交换, 传递信息, 参与有丝分裂等重要功能^[5, 6]^。NSCLC中βⅢ-tubulin表达与肿瘤的分化程度和分级有关, 表达越高, 肿瘤的分化越差、分级越高, 并且肿瘤转移潜力增加^[7, 8]^。研究^[9]^发现NSCLC在腺癌组织中的βⅢ-tubulin表达水平较鳞癌和其它组织学类型高。

β-tubulin是抗微管药物的主要细胞靶点。其中βⅢ-tubulin高表达NSCLC细胞株和卵巢癌细胞株的耐药有关^[10, 11]^。RNA干扰(RNA interference, RNAi)在探索基因功能、基因治疗、病毒性疾病、传染性疾病以及肿瘤的治疗领域有广泛应用。与传统反义核酸进行转录后基因沉默相比, 能高效地特异性抑制目的基因。设计更简便、作用迅速、效果明显, 能根据不同病情, 设计个体化治疗方案。

本研究利用RNA干扰技术沉默人肺腺癌A549/Taxol细胞中*βⅢ-tubulin*基因的表达, 探讨靶基因下调后对化疗药物紫杉醇的敏感性的变化以及对细胞周期和细胞凋亡的影响。

## 1 材料与方法

### 1.1 细胞株及主要试剂

人肺腺癌耐紫杉醇A549细胞株(A549/Taxol)来自山东省肿瘤医院中心实验室, A549/Taxol在0.2 μg/mL的Taxol中正常生长。DMEM培养基(Hyclone公司), OPTI-MEM培养液(GIBCO公司), 胎牛血清(杭州四季青生物制品公司); βⅢ-tubulin siRNA序列(上海吉玛公司), PCR引物(上海生物工程公司); RT-PCR试剂:Lipofectamine 2000、Trizol试剂(invitrogen公司); 反转录试剂盒Script^TM^ RT reagent kit以及SYBR Premix Ex Taq^TM^(Perfect Real Time)(TaKaRa公司); Western blot试剂:βⅢ-tubulin鼠抗人单抗(Abcam公司), β-actin鼠抗人单抗(Amersham Biosciences公司), 羊抗人二抗(中杉公司); 紫杉醇(paclitaxel)(四川太极制药有限公司), -20 ℃冻存。

### 1.2 细胞培养

人肺腺癌A549/Taxol细胞株在含10%胎牛血清的DMEM培养基, 37 ℃、5%的CO_2_培养箱中培养, 隔天换液, 取对数生长期的细胞进行实验。

### 1.3 人工合成βⅢ-tubulin siRNA序列

根据Genebank βⅢ-tubulin mRNA序列和siRNA的设计原则, 人工设计合成3对βⅢ-tubulin siRNA序列和1对阴性对照siRNA序列:βⅢ-tubulin siRNA sense 5’-UCUCUUCAGGCCUGACAAUTT-3’, antisense 5’-AUUGUCAGGCCUGAAGAGATT-3’; βⅢ-tubulinsiRNA sense 5’-GACCUCAACCACCUGGUAUTT-3’, antisense 5’-AUACCAGGUGGUUGAGGUCTT-3’; βⅢ-tubulin siRNA sense 5’-GCACGUUGCUCAUCAGCAATT-3’, antisense 5’ UUGCUGAUGAGCAACGUGCTT-3’; Negative control siRNA sense 5’-UUCUCCGAACGUGUCACGUTT-3’, antisense 5’-ACGUGACACGUUCGGAGAATT-3’。

### 1.4 细胞转染

采用阳性脂质体Lipofectamine^TM^2000介导转染。转染前一天将对数生长期的A549/Taxol细胞接种于6孔板, 调整细胞密度为1×10^5^/mL, 设siRNA组:(βⅢ-tubulin siRNA-1组、βⅢ-tubulin siRNA-2组、βⅢ-tubulin siRNA-3组)、阴性对照组(Negative control siRNA)、mock组(只含转染试剂)和空白对照组(non-transfection)。将6孔板置于37 ℃、5%CO_2_孵箱中培养。次日, 待细胞生长至70%-80%融合时, 按说明书进行转染。转染5 h后更换为全培养液。

### 1.5 实时荧光定量RT-PCR检测βⅢ-tubulin mRNA表达

转染48 h后TRIzol法提取细胞内总RNA, 参照TRIzol Reagent说明书分别提取6组细胞内总RNA, 检测总RNA纯度及浓度。反转录合成cDNA第一链, 体系如下:5×Prime Script^TM^ Buffer 2 μL, Prime Script^TM^ RT Enzyme Mix Ⅰ 0.5 μL, Oligo dT Primer (50 μM) 0.5 μL, Random 6 mers (100 μM) 0.5 μL, Total RNA 500 ng, Rnase free dH_2_O至10 μL。反应条件:37 ℃ 15 min, 85 ℃ 5 s。对各组细胞反转录合成的βⅢ-tubulin cDNA模板进行PCR扩增反应, β-actin为内对照。引物序列如下:βⅢ-tubulin:Forward primer 5'-GGAGATCGTGCACATCCAG-3', Reverse primer 5'-TCGATGCCATGCTCATCAC-3';β-actin:Forward primer 5'-TGGCACCCAGCACAATGAA-3', Reverse primer 5'-CTAAGTCATAGTCCGCCTAGAAGCA-3'。实时荧光qPCR反应体系(50 μL)如下:SYBR^®^ Premix Ex Taq^TM^(2×)25.0 μL, PCR Forward Primer(10 μM) 1.0 μL, PCR Reverse Primer(10 μM)1.0 μL, ROX Reference Dye(50×), 1.0 μL, DNA模板4.0 μL, dH_2_O(灭菌蒸馏水)18.0 μL, Total 50.0 μL。扩增反应条件:95 ℃ 10 s 1个循环, 95 ℃ 5 s 40个循环, 60 ℃ 31 s 40个循环。

扩增结束后excell软件分析计算Ct值、△Ct、△△Ct、2^-△△Ct^, △Ct=CtβⅢ-tubulin-Ctβ-actin, △△Ct=实验组△Ct-对照组△Ct, 并计算βⅢ-tubulin mRNA含量。

### 1.6 Western blot检测βⅢ-tubulin蛋白表达

用RT-PCR法筛选的有效βⅢ-tubulin siRNA-1序列6孔板转染细胞, 设对照组。48 h后, 提取细胞总蛋白, 测定总蛋白浓度, 蛋白质变性; 电泳分离蛋白质; 转膜将蛋白质从凝胶转移到PVDF膜; 4 ℃封闭过夜; 加入βⅢ-tubulin鼠抗人一抗(1:2, 000)、β-actin一抗(1:2, 000), 室温孵育2 h, 洗膜3次10 min/次; 加入羊抗人二抗(1:2, 000), 室温孵育2 h, T洗膜3次10 min/次; 显色; 曝光; 常规显影定影; Bio2rad图像分析系统进行图像分析。β-actin为内对照。

### 1.7 MTT法检测紫杉醇对A549/Taxol细胞的敏感性

转染48 h后, 收集处于对数生长期的细胞, 制成为1×10^5^/mL的细胞悬液, 接种于96孔培养板中, 每孔100 μL, 紫杉醇设6个浓度倍比稀释等级, 每浓度设3个复孔。待细胞贴壁后加入不同浓度Taxol(1.25 μg/mL、2.5 μg/mL、5.0 μg/mL、10.0 μg/mL、20.0 μg/mL、40.0 μg/mL)。于37 ℃、5%CO_2_孵箱中培养48 h; 加入MTT溶液(5 mg/mL)每孔10 μL, 继续培养4 h; 加10%SDS-HCL每孔100 μL; 24 h后用Bio-rad 450型酶标仪测各孔吸光度(*A*), 检测波长为570 nm, 参考波长为630 nm。按下式计算各浓度条件下细胞抑制率(inhibitory rate, IR), IC=(1-实验组*A*值/对照组*A*值)×100%。Excell软件绘制浓度效应曲线, 实验重复3次。

### 1.8 流式细胞仪检测细胞凋亡(Annexin V-FITC法)

Taxol诱导处理细胞48 h后, 制备细胞悬液, PBS溶液洗涤2次, 1, 000 rpm离心5 min, PBS溶液重悬细胞调整细胞密度为1×10^6^/mL, 按照Annexin V-FITC细胞凋亡检测试剂盒说明操作, 流式细胞仪检测细胞早期凋亡率。

### 1.9 流式细胞仪检测细胞周期

Taxol诱导处理细胞48 h后, 制备细胞悬液, PBS溶液洗涤, 1, 000 rpm离心5 min, PBS溶液重悬细胞调整细胞密度为1×10^6^/mL, 1, 000 r离心5 min, 弃去PBS, 加入预冷70%乙醇固定, 4 ℃过夜。加入碘化丙啶缓冲液500 μL/孔, 4 ℃避光30 min, 流式细胞仪检测细胞周期。

### 1.10 统计学分析

实验数据以Mean±SD表示, 采用SPSS 17.0统计软件进行*t*检验, *P* < 0.05表示差异有统计学意义。

## 2 结果

### 2.1 βⅢ-tubulin siRNA转染下调A549/Taxol细胞中βⅢ-tubulin mRNA表达

与NC siRNA组、mock、non-transfection组比较, βⅢ-tubulin siRNA-1、βⅢ-tubulin siRNA-2、βⅢ-tubulin siRNA-3组的mRNA表达水平均有不同程度的下调(*P* < 0.05), 其中βⅢ-tubulin siRNA-1组的抑制率最高, 为(87.73±4.87)%(*P* < 0.01)(图 1)。

**1:**
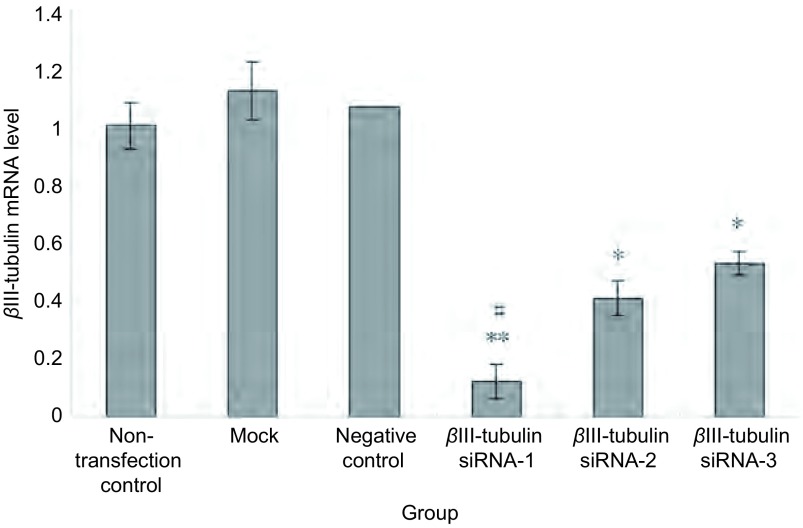
转染后A549/Taxol细胞中*β*Ⅲ-tubulin mRNA含量。^*^:与对照组相比, *P* < 0.05;#:与*β*Ⅲ-tubulin siRNA-2、*β*Ⅲ-tubulin siRNA-3组相比, *P* < 0.05。*β*Ⅲ-tubulin siRNA-1组的抑制率最高(87.73±4.87)%, 与对照组比较, 差异有统计学意义(*P* < 0.01)。 Expression of *β*Ⅲ-tubulin mRNA in A549/Taxol after transfection detected by qRT-PCR.^*^:compared with the control, *P* < 0.05;^**^:compared with the control, *P* < 0.01;#:compared with *β*Ⅲ-tubulin siRNA-2, *β*Ⅲ-tubulin siRNA-3, *P* < 0.05.*β*Ⅲ-tubulin siRNA-1 sequence showed the highest transfection efficiency, (87.73±4.87)%.The difference was statistically significant compared with control.

### 2.2 βⅢ-tubulin siRNA转染下调A549/Taxol细胞中βⅢ-tubulin蛋白表达

Western blot结果显示:βⅢ-tubulin siRNA组可见到分子量为51 kDa左右微弱的βⅢ-tubulin特异性条带和β-actin条带, 其靶蛋白表达量较对照组明显减少, 而β-actin条带与对照组基本一致。这与βⅢ-tubulin mRNA表达减少结果相一致(图 2)。

**2:**
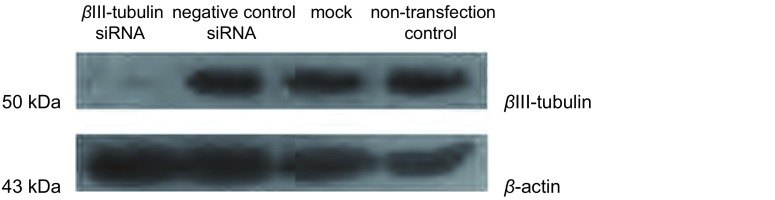
Western blot检测蛋白的*β*Ⅲ-tubulin表达。与对照组相比, *β*Ⅲ-tubulin siRNA-1组靶蛋白表达水平明显减少。 Expression of *β*Ⅲ-tubulin protein level detected by Western blot.Compared with the control group, protein expression of *β*Ⅲ-tubulin siRNA-1 after transfection was significantly reduced.

### 2.3 βⅢ-tubulin下调增加A549/Taxol细胞对紫杉醇的敏感性

MTT法检测结果显示, βⅢ-tubulin siRNA转染A549/Taxol细胞后, 紫杉醇对βⅢ-tubulin siRNA组的细胞抑制率明显高于对照组(51.77±4.60)%(*P* < 0.01), 且20 μg/mL时抑制率明显(图 3)。

**3:**
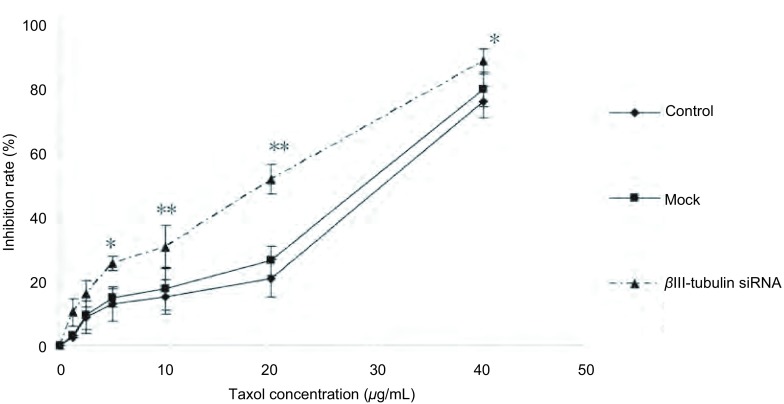
MTT法检测紫杉醇处理转染组后细胞抑制率曲线。转染组细胞抑制率较对照组增加(*P* < 0.01), 且10 *μ*g/mL、20 *μ*g/mL时, 抑制率最明显(*P* < 0.01)。^**^P < 0.01, ^*^P < 0.05。 Inhibition ratio assays on *β*Ⅲ-tubulin siRNA induced by paclitaxel.The inhibition ratio of *β*Ⅲ-tubulin siRNA group was higher than that of control group (*P* < 0.05).And it has obviously increased with 10 *μ*g/mL and 20 *μ*g/mL (*P* < 0.01).^**^P < 0.01, ^*^P < 0.05.

### 2.4 A549/Taxol细胞βⅢ-tubulin下调增加紫杉醇诱导的细胞凋亡

流式细胞仪检测细胞凋亡, 结果显示紫杉醇作用后βⅢ-tubulin siRNA组细胞早期凋亡率较对照组明显增加(*P* < 0.05), 其中Taxol为20 μg/L最明显, 两组的早期凋亡率分别为(40.12±3.86)%、(21.47±5.44)%, 有统计学差异(*P* < 0.01)(图 4)。

**4:**
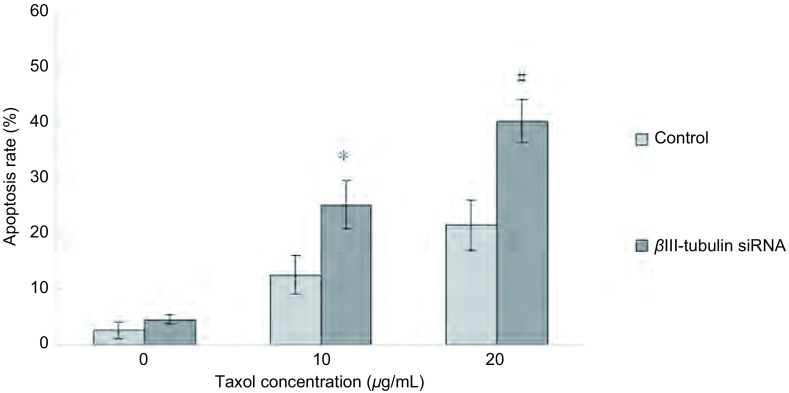
紫杉醇诱导转染后A549/Taxol细胞的早期凋亡率。转染组细胞早期凋亡率较对照组增加(*P* < 0.05)。且20 *μ*g/mL时抑制率明显(*P* < 0.01)。 Apoptosis ratio induced by taxol on A549/Taxol transfected by *β*Ⅲ-tubulin siRNA.The early apoptosis rate of transfected group were higher than that of control group (*P* < 0.05).And it has the significantly increased in 20 *μ*g/mL (*P* < 0.01).#*P* < 0.01, ^*^P < 0.05.

### 2.5 βⅢ-tubulin siRNA转染后检测紫杉醇对A549/Taxol细胞的细胞周期影响

细胞周期检测结果显示, 紫杉醇处理细胞组的G_2_/M期细胞百分率高于对照组, 差异有统计学意义(*P* < 0.05), 表明紫杉醇将细胞阻滞在G_2_/M期, 可能与诱导细胞凋亡有关, 且紫杉醇处理转染组后细胞晚期凋亡率较对照组增加(图 5)。

**5:**
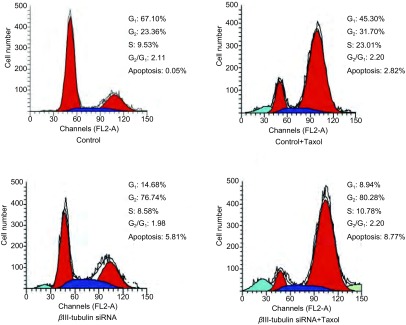
紫杉醇处理后各细胞组细胞周期分析。紫杉醇处理组G_2_-M期细胞比例较未处理组明显增高(*P* < 0.05)。且*β*Ⅲ-tubulin siRNA+Taxol组细胞晚期凋亡率较对照组增加。 Cell cycle assays of A549/Taxol on effect of taxol.G_2_/M content induced by paclitaxel is obviously increased than untreated samples.And the apoptosis rate of *β*Ⅲ-tubulin siRNA+Taxol group were higher than that of control group.

## 3 讨论

肿瘤耐药是影响肺癌患者化疗疗效的主要因素。影响NSCLC耐药的因素主要有多药耐药(multi-drug resistant, *MDR*)基因、多药耐药相关蛋白(multi-drug resistant related protein, *MRP*)基因及它们产物表达的增加, 以及拓扑异构酶Ⅱ(Topoisomerase Ⅱ, Topo Ⅱ)表达下降和谷胱甘肽(Glutathione, GSH)及谷胱甘肽S转移酶(glutathione S-transferase, GST)系统表达增加。此外研究^[12]^表明, 细胞信号转导中相关因子的表达异常、肿瘤细胞DNA修复的异常及其他相关基因的表达异常与肺癌耐药的产生也存在密切联系。因此筛选有效的分子指标, 以预测患者对药物的敏感性, 可以更好的指导个体化治疗、提高化疗疗效。

微管是细胞骨架的重要构成成分, 对维持细胞的空间结构以及各种生理活动都具有重要作用。微管是由α-tubulin和β-tubulin形成的异二聚体组装成的多聚体, 其中有6种β微管蛋白同型体, 而βⅢ-tubulin与抗微管类化疗药物敏感性有最密切的关系^[6]^。当可溶性微管蛋白水平增加后, β-tubulin mRNAs翻译水平出现下调, 机体通过自身调控机制调节β-tubulin同型体的表达^[13, 14]^。抗微管蛋白聚合药物有三大类, 即秋水仙碱类、鬼臼素类和长春花生物碱类(如长春瑞滨)。而促进微管聚合、抑制微管蛋白解聚的药物目前有紫杉醇和多西紫杉醇两类^[15]^。

β-tubulin是抗微管类药物的主要靶点, 分析β-tubulin同型体的表达确定药物耐药有关的具体靶蛋白是非常有必要的。研究^[16, 17]^表明βⅢ-tubulin高表达与作用于微管的药物有关, 而与其他β-tubulin亚型没有交叉作用。

研究^[18.19]^表明βⅢ-tubulin低表达的NSCLC患者经紫杉类化疗其疗效及预后均优于高表达者。Vilmar等^[9]^的研究认为NSCLC中不同组织学类型其βⅢ-tubulin表达不同, 腺癌的阳性表达率较鳞癌和其它组织学类型的阳性表达率高, βⅢ-tubulin-negative腺癌患者较βⅢ-tubulin-positive腺癌者PFS和OS延长(*P* < 0.05), 且其OS较βⅢ-tubulin-negative鳞癌或大细胞癌患者延长(*P* < 0.05)。

许多临床前研究^[5, 12, 21]^和临床证据表明βⅢ-tubulin在肿瘤的发生、发展中发挥重要作用。研究发现上皮源性肿瘤包括NSCLC中βⅢ-tubulin表达异常与肿瘤分化能力、侵袭性密切相关, 其高表达者表现为肿瘤低分化、恶性程度高、侵袭性增加等特点, 且患者生存期短、预后差, 故不仅是化疗耐药的标记物也是NSCLC的独立预后因子^[20]^。

许多临床研究发现肺癌患者术后肿瘤组织中βⅢ-tubulin阳性表达组的OS短于阴性表达组, 两者有统计学差异(*P* < 0.05), 证明βⅢ-tubulin可作为NSCLC术后的一个独立预后因子, βⅢ-tubulin高表达可能是肺癌的预后因子。Rosell等^[18]^和王峻等^[12]^的研究显示在NSCLC肿瘤组织中βⅢ-tubulin高表达对长春碱类药物敏感性低。Vilmar等^[9]^的研究认为βⅢ-tubulin-negative的晚期肺腺癌患者较βⅢ-tubulin-positive腺癌者PFS和OS延长(*P* < 0.05), 并且化疗后其OS较鳞癌或大细胞癌者延长(*P* < 0.05)。以上研究表明βⅢ-tubulin高表达的患者预后差, PFS和OS短, 而且高表达者对化疗耐药, 可能与肿瘤的进展有关。

本研究应用βⅢ-tubulin siRNA转染A549/Taxol细胞, 并检测βⅢ-tubulin基因和蛋白水平的表达情况, 并检测靶基因下调后对紫杉醇的敏感性的变化以及细胞凋亡和细胞周期情况。

结果显示βⅢ-tubulin siRNA组的靶基因表达明显下调(87.73±4.87)%(*P* < 0.01);βⅢ-tubulin siRNA组的靶蛋白表达较对照组明显减少, 与βⅢ-tubulin mRNA表达下调相一致; 紫杉醇处理后βⅢ-tubulin siRNA组的细胞抑制率较control组明显增加(51.77±4.60)%(*P* < 0.01), 表明下调*βⅢ-tubulin*基因表达明显增加对紫杉醇的敏感性。细胞早期凋亡率较对照组明显增加(*P* < 0.01), 且20 μg/mL时明显(40.12±3.86)% *vs* (21.47±5.44)%, 提示下调βⅢ-tubulin后紫杉醇诱导的细胞早期凋亡率增加, 抑制肿瘤增殖; 紫杉醇诱导后βⅢ-tubulin siRNA组G_2_/M期细胞百分率明显高于未处理组(*P* < 0.05), 将细胞阻滞在G_2_/M期, 抑制肿瘤细胞增生, 可能与促进细胞凋亡有关。且紫杉醇诱导转染组细胞晚期凋亡率较对照组增加, 差异无统计学差异, 细胞早期凋亡率明显增加。因此βⅢ-tubulin可能是在调节化疗药物反应方面的一个重要的生存反应因子。

Hasegawa等^[22]^利用反义寡核苷酸干扰A549/Taxol下调靶基因表达后细胞对紫杉醇的敏感性较对照组明显增加(*P* < 0.05)。Gan等^[16]^利用siRNA干扰两组肺癌细胞株NCI-H460和Calu-6, 发现βⅢ-tubulin siRNA干扰细胞后Ⅰ型、Ⅱ型、Ⅳ型和总β-tubulin表达均较对照组无差异。而βⅢ-tubulin表达水平较对照组明显减少。且两组细胞对作用于微管的两种化疗药物紫杉醇和长春新碱的药物敏感性较对照组明显增加。细胞凋亡率较对照组明显增加。这与本研究结果相一致。证实了βⅢ-tubulin高表达对紫杉醇耐药, 相反低表达对紫杉醇敏感, 通过增加细胞凋亡来抑制肿瘤增殖。

βⅢ-tubulin表达与紫杉醇敏感性有关, 体内和体外实验已经证实低表达者疗效及预后均优于高表达者, 提示βⅢ-tubulin有可能作为预测紫杉醇敏感性的一个指标, 有利于指导NSCLC的临床个体化治疗。
